# Polyneuritis Preceded by Inflammation of the Parotid, Lachrymal and Mammary Glands

**Published:** 1926

**Authors:** Beatrice Rogers, J. Hervey Bodman

**Affiliations:** Honorary Medical Officer, Bristol Maternity Hospital; Physician, Bristol Homæopathic Hospital


### POLYNEURITIS PRECEDED BY INFLAMMATION OF THE PAROTID, LACHRYMAL AND MAMMARY GLANDS.

BY

Beatrice Rogers, L.R.C.P., L.R.C.S. Edin.,
*Honorary Medical Officer, Bristol Maternity Hospital,*

AND

J. Hervey Bodman, M.D. Lond.,
*Physician, Bristol Homœopathic Hospital.*

---

The case which we report is that of a lady, aged 61, whose health had been good except for a liability to migrainous headaches. These had been treated at a foreign clinic at the end of 1924 by means of mistletoe, male fern and similar remedies, with good result.

In April, 1925, she developed acute pharyngitis without fever. This was followed by parotitis on the right side, which subsided without suppuration, leaving the gland slightly swollen for some months. During its subsidence the left parotid became swollen, but this resolved more quickly. In May a similar inflammation attacked the lachrymal glands, first the left and then the right. This lasted for about six weeks, and left no permanent swelling. In July the whole left breast became very swollen and tender but did not suppurate. Even as late as October a mass as large as a walnut could still be felt in the breast.

In August the second phase of the malady began, with sensory symptoms in the right gluteal region and the tip of the left forefinger. Within a few weeks sensation was impaired in the sole of the right foot, and the right ankle was weak. On October 18th the right big toe, the right gluteal region and the tip of the left forefinger were noted to be insensitive to pain, and numbness with a feeling of pins and needles was found in the distal parts of the right leg and left arm. The knee jerks were present, and the plantar responses flexor. There was a trace of albumin in the urine. The pain in the right leg and foot increased to such a pitch as to prevent sleep, and the weakness and analgesia also increased. In November the side of the left foot became numb and patches of erythema were observed over the abdomen and the right shin. More than once attacks of diarrhœa and vomiting were observed. At this time, also, reliable information as to the dosage of the drugs prescribed in the foreign clinic was secured. Hitherto some hope of referring the symptoms to the use of these drugs had been entertained. A little support was lent to this view by papers by Magnus-Levy[1] and others, which seemed to show that the persistent exhibition of male fern might provoke symptoms something like those of ergotism. The doses were, however, much too small to be held responsible, and there remained no alternative but to ascribe the whole illness to some obscure infection, first of a series of glands and later of the central nervous system.

In December she had very little sleep; was very restless and irritable at night; and often mildly delirious, holding conversations with persons supposed to be present, sometimes in English, sometimes in German. She would suddenly fall asleep for a few minutes, even in the middle of a conversation or of a meal. On December 5th the general nutrition was good, and the following notes of the nervous state were made. No wasting of muscles observed. No trophic changes in skin. Flaccid paralysis of most of muscles of both lower extremities, more marked in distal portion of each limb. Foot-drop very marked on right side. Knee jerks: Right, present; left, absent. Plantar reflexes: no response obtained. Flaccid paralysis of muscles of left forearm and hand; marked wrist-drop on left side. Most movements of right hand and forearm are possible, but much reduced in power. Right triceps jerk present; left, absent. Upper abdominal reflexes absent. Lower abdominal reflexes present. Irregular areas of defective sensation in all four extremities, more marked in distal parts. Pupillary reactions and ocular fundi normal. Pupillary border of iris slightly irregular owing to minute projections of brown pigment.

There was much nasal obstruction, causing her discomfort when awake and snoring during sleep; and a moderate amount of muco-purulent nasal discharge. Two or three small areas of induration of the skin, with slight redness, one over left breast and another on one arm, were noted. The urine contained a faint trace of albumin. The weakness of the right hand and forearm increased so that the patient could no longer use her handkerchief or feed herself. A slight improvement took place in the paralysis of the legs. The delirium at night became more noisy, and was accompanied by active attempts to get out of bed and by antipathy to her nurse.

The following pathological report was furnished by Dr. G. Hadfield on December 15th:—

*Blood.*—Wassermann reaction negative. Van den Bergh's reaction negative. Blood urea, 69 mgm. per 100 c.cs.

*Urine.*—Sp. gr. 1020. Acid reaction. Albumin, 0·01 per cent. No sugar. Urea, 2·3 per cent. Excess of leucocytes. Moderate number of hyaline casts. Probably a slight catarrhal nephritis is present.

*Stool.*—Nothing abnormal in cultures. Occult blood test negative.

On December 16th the patient was seen by Dr. James Collier, who diagnosed the case as uveo-parotitic paralysis. He performed a lumbar puncture. The cerebro-spinal fluid appeared normal to the naked eye, and the rate of flow was normal. The following is a report on the fluid by Dr. J. G. Greenfield :—

*Cerebro-spinal fluid.*—Clear, colourless. Undue froth. No coagulum.

*Cells.*—2 per c.m., small mononuclears.

*Total protein.*—0·1 per cent. (three to four times normal).

*Globulin.*—Nonne Apelt reaction weakly positive. Pandy reaction positive.

*Lange's Gold Sol. Test.*—·0000001221 (late curve due to protein excess).

*Wassermann reaction.*—Negative.

The only abnormality in this fluid is the increase of protein, which is not infrequent in cases of neuritis.

About twenty-four hours after the lumbar puncture she began to have severe headache, which lasted for nearly twenty-four hours and was accompanied by repeated vomiting, usually of the cerebral type, effortless and without retching. During the next fortnight the mental and paralytic symptoms remained much the same. Occasionally the pulse became feeble and the patient's colour rather ashy, causing considerable anxiety to the nurse for a short time.

About the same time the nurse reported that incontinence of urine had become established, and that there were sometimes jerky movements of the right arm and left leg and sometimes of the whole trunk. Also occasional opisthotonos was observed, the back being completely off the bed at these times. From December 17th to January 2nd the patient continued to have occasional turns of vomiting. The temperature was never raised, and was usually rather subnormal. During the latter part of December there was slight conjunctivitis with slight muco-purulent discharge and redness of margins of lids. A swab was taken of the discharge and was examined by Dr. Hadfield, who reported that films showed few leucocytes and only a few micro-organisms ; a culture yielded *Staphylococcus Albus* only.

During the first two weeks of January the mental condition improved to some extent and there was less delirium ; but there was a gradual loss of physical strength and at times the pulse was decidedly feeble. The nasal obstruction continued troublesome, and examination showed that both maxillary antra and the right frontal sinus were dark on transillumination. The question of operation was carefully considered, and it was felt that there was just a possibility that the draining of the infected sinuses might give the patient a chance of recovery : in this view Dr. James Collier, who was consulted by letter, concurred. On January 20th both maxillary antra were opened by Mr. C. Osmond Bodman through the inferior meatus of the nose, and were found to contain pus. A free opening was made to ensure drainage and to permit of irrigation. The right frontal sinus was washed out and was found to contain pus, and part of the right middle turbinal was removed to ensure better drainage.

During the days following the operation the breathing through the nose was much more free than before, but the patient continued to grow weaker, and there was some bronchial catarrh. There was no pyrexia, and no sign of any intracranial complication. Death took place on January 24th, apparently as the result of progressive enfeeblement of the myocardium. Consciousness was retained until a short time before the end came. No autopsy was performed.

Certain features of the case narrated above appear to call for brief comment. Although included in Dr. George Parker's table of cases of uveo-parotitic paralysis, this case differs from most of the other cases hitherto reported in two important particulars. In the first place it must be pointed out that at no time during the period in which the patient was under our observation was there anything approaching a definite uveitis or iridocyclitis. In most of the other recorded cases this was a well-marked feature at some period in the course of the illness, and in some it was of such intensity as to cause serious loss of visual acuity for many weeks or months. In our case there was a history of an ill-defined attack of pain in one eye, and at the time the case was diagnosed as uveo-parotitic paralysis it was thought that this attack had occurred during the first stage of the present illness, but it was ascertained subsequently that it was more than two years before the earliest symptoms of the present illness. When the eyes were examined early in December there was no keratitis punctata and there were no posterior synechiæ. The only abnormality noted was a slight irregularity of the pupillary border of the iris.

The only feature in which our case differed from nearly all the other cases appearing in the literature under the title of uveo-parotitic paralysis, or some similar appellation, was the way in which the morbid process involved the functions of the brain and of the heart. For several weeks there was much mental derangement, especially at night; and during the last few weeks there were threatening indications of cardiac failure, presumably due to the action of a toxin on the heart. It is this latter feature that appears to have been the cause of the fatal issue.

In conclusion, the heavily-infected condition of some of the nasal accessory sinuses must be noted as probably having some bearing upon the etiology of the case.

REFERENCE.

[1] Magnus-Levy, *Berl. Klin. Woch.*, 1911, March 27th.

